# A Scoping Review of Flow Research

**DOI:** 10.3389/fpsyg.2022.815665

**Published:** 2022-04-07

**Authors:** Corinna Peifer, Gina Wolters, László Harmat, Jean Heutte, Jasmine Tan, Teresa Freire, Dionísia Tavares, Carla Fonte, Frans Orsted Andersen, Jef van den Hout, Milija Šimleša, Linda Pola, Lucia Ceja, Stefano Triberti

**Affiliations:** ^1^Department of Psychology, University of Lübeck, Lübeck, Germany; ^2^Faculty of Psychology, Ruhr-Universität Bochum, Bochum, Germany; ^3^Department of Psychology, Linnaeus University, Växjö, Sweden; ^4^ULR 4354 - CIREL - Centre Interuniversitaire de Recherche en Education de Lille, Université de Lille, Lille, France; ^5^Department of Psychology, Goldsmiths University of London, London, United Kingdom; ^6^School of Psychology, University of Minho, Braga, Portugal; ^7^Faculty of Human and Social Sciences, University Fernando Pessoa, Porto, Portugal; ^8^Department of Education, University of Aarhus, Aarhus, Denmark; ^9^Industrial Engineering and Innovation Sciences, Eindhoven University of Technology, Eindhoven, Netherlands; ^10^Institute of Psychology Henri Pieron, Université Paris 5 René Descartes, Paris, France; ^11^Department of Cultural Heritage and Environment, University of Milan, Milan, Italy; ^12^IESE Business School, University of Navarra, Barcelona, Spain; ^13^Department of Oncology and Hemato-Oncology, University of Milan, Milan, Italy

**Keywords:** flow, scoping review, individual level, contextual level, cultural level

## Abstract

Flow is a gratifying state of deep involvement and absorption that individuals report when facing a challenging activity and they perceive adequate abilities to cope with it (EFRN, 2014). The flow concept was introduced by Csikszentmihalyi in 1975, and interest in flow research is growing. However, to our best knowledge, no scoping review exists that takes a systematic look at studies on flow which were published between the years 2000 and 2016. Overall, 252 studies have been included in this review. Our review (1) provides a framework to cluster flow research, (2) gives a systematic overview about existing studies and their findings, and (3) provides an overview about implications for future research. The provided framework consists of three levels of flow research. In the first “Individual” level are the categories for personality, motivation, physiology, emotion, cognition, and behavior. The second “Contextual” level contains the categories for contextual and interindividual factors and the third “Cultural” level contains cultural factors that relate to flow. Using our framework, we systematically present the findings for each category. While flow research has made progress in understanding flow, in the future, more experimental and longitudinal studies are needed to gain deeper insights into the causal structure of flow and its antecedents and consequences.

## Introduction

Flow “is a gratifying state of deep involvement and absorption that individuals report when facing a challenging activity and they perceive adequate abilities to cope with it” (EFRN, 2014). The phenomenon was described by Csikszentmihalyi (1975) in order to explain why people perform activities for no reason but for the activity itself, without extrinsic rewards. During flow, people are deeply motivated to persist in their activities and to perform such activities again (Csikszentmihalyi, 1975; EFRN, 2014). Csikszentmihalyi (1975, 1990) distinguished up to nine characteristics of the flow experience: (1) challenge-skill-balance, (2) merging of action and awareness, (3) clear goals, (4) unambiguous feedback, (5) concentration on the task, (6) sense of control, (7) loss of self-consciousness, (8) time transformation, and (9) autotelic experience.

The first of these characteristics—the challenge-skill balance—gained much attention in flow research. In his Flow Channel Model, Csikszentmihalyi (1975) operationalized flow in the context of skills and challenges: if the individual’s skills meet the situational challenges, the individual is in the so-called *flow channel* and flow occurs. In later modifications of this model, as in the Experience Fluctuation Model (EFM), flow was said to occur if both challenges and skills are *high* and in balance (e.g., Massimini et al., 1987; Carli et al., 1988; Csikszentmihalyi, 1997). This assumption gained empirical support: for example, Inkinen et al. (2014) showed that if challenges and skills are high and in balance, this combination is characterized by an active and pleasant emotional experience, as described in the EFM. Also, a recent meta-analytical study confirmed the stability of challenge-skill balance as a condition of flow (Fong et al., 2015), together with clear goals and sense of control.

Later, Nakamura and Csikszentmihalyi (2002) and Landhäußer and Keller (2012) sorted Csikszentmihalyi’s (1990) characteristics of flow experience into preconditions and components of flow. They also defined the balance between task demands and skills as a central precondition of flow, together with clear goals and clear feedback. They defined components of flow as concentration, merging of action and awareness, sense of control, autotelic experience, reduced self-consciousness, and transformation of time. Further conceptualizations of flow exist (e.g., Bakker, 2005; Engeser and Rheinberg, 2008; Abuhamdeh, 2021; Barthelmäs and Keller, 2021; for an overview see Engeser et al., 2021; Peifer and Engeser, 2021). Recently, Peifer and Engeser (2021) have critically discussed the existing components of flow and proposed an integration of those into the three meta-components *absorption*, *perceived demand-skill balance*, and *enjoyment*.

Since the introduction of the flow concept, there has been much research investigating the concept itself, its preconditions, and its consequences. The research shows that “flow experiences can have far-reaching implications in supporting individuals’ growth, by contributing both to personal wellbeing and full functioning in everyday life” (EFRN, 2014). Potentially due to its positive consequences, flow research is further growing and there is a wealth of empirical articles dedicated to this phenomenon. However, due to the large amount of studies, there is a lack of a broad and systematic overview on flow research. Accordingly, this review aims to provide such a structured overview of flow research and to provide directions for future flow research.

Since 2012, the European Flow-Researchers’ Network (EFRN) has met on a yearly basis to foster scientific progress in flow research and application. Following this aim and having identified the described lack of agreement within flow research, the network decided in their meeting in November 2015 to unite their expertise and provide a scoping review on studies addressing flow experience published as of the year 2000. The advantage of a scoping review is that it collects, evaluates and presents the available research with a more systematic approach than is used in traditional review articles (Arksey and O’Malley, 2005). Compared to meta-analyses or systematic reviews, a scoping review regards not just a specific, narrow research question, but a broad scope of research with respect to a certain concept (Arksey and O’Malley, 2005), in our case, flow experience. Accordingly, a scoping review aims to identify and structure existing research in order to provide a framework and to build a basis for future research.

The scoping review follows three steps: first, we present a framework to structure flow research. Second, we review empirical flow research that has been published between 2000 and 2016. Third, based on our results, we discuss implications for future research.

### Framework to Structure Flow Research

**FIGURE 1:**
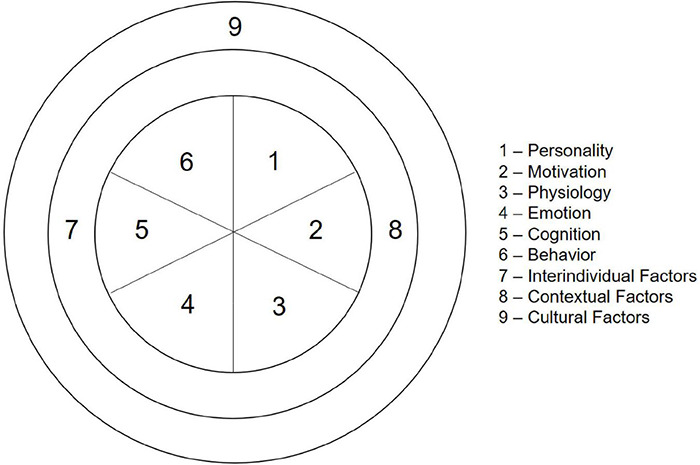
Categorization of flow research 2000–2016.

In order to structure and review the empirical research regarding flow experiences, the authors developed a framework (see Figure 1). The framework consists of three circles lying within each other and containing categories of flow research. The inner circle represents individual factors. On this individual level, we distinguish between the categories of personality, motivation, physiology, emotion, cognition, and behavior. The middle circle—the contextual level—represents the categories contextual and interindividual factors and the outer circle represents the cultural category. Within our framework, all categories contain preconditions or consequences of flow, and all categories can influence each other.

## Methods

As proposed by Arksey and O’Malley (2005), our scoping review was developed using the following 6 steps.

### Identification of the Research Question

The importance of providing a scoping review on flow experience was identified during the 4th meeting of the European Flow Researchers’ Network (EFRN) in Braga (Portugal), 2015. To fulfill this aim, the network searched for a systematic overview of the existing flow research as a basis for future research. The finding of that literature search was that the number of publications on flow experience is growing, but that a systematic overview was not available. Accordingly, the EFRN decided to unite their expertise to develop such a systematic overview, i.e., a scoping review. To start, during the 4th EFRN meeting in Braga (Portugal), EFRN members worked on a preliminary framework to categorize flow research.

### Literature Research

For the literature search, we consulted the platforms *PsycInfo, PubMed, PubPsych, Web of Science* and *Scopus*. We searched for empirical studies using the terms “*flow/optimal experience/challenge-skill balance”* in order to cover different terms for flow which are typically used in the literature. Also, we excluded *“cerebral blood flow”* and *“work-flow centrality,”* as these terms produces many false hits. Further, we decided to add the term “*Csikszentmihalyi*” to the search, as we considered that reputable articles on flow would cite Csikszentmihalyi and, at the same time, many articles which are not related to flow experience would be excluded. We only included empirical studies that were published between 2000 and 2016. The resulting search string was (for PsycInfo):

(((“flow” or “optimal experience” or “challenge-skill balance”) and “Csikszentmihalyi”) not “cerebral blood flow” not “work-flow centrality”).af. and (“2000” or “2001” or “2002” or “2003” or “2004” or “2005” or “2006” or “2007” or “2008” or “2009” or “2010” or “2011” or “2012” or “2013” or “2014” or “2015” or “2016”).yr.

We did not include conference abstracts or articles that were not in the English language. Also, within this first step, we excluded publications that clearly did not deal with the topic of flow experience. The literature search was conducted in 2016 and updated in 2017 to cover also the full year of 2016.

### Selection of Relevant Studies

Overall, we found 257 publications that were then rated by the authors with respect to their relevance for our scoping review. In the next step, publications were excluded if they did not contain original data on flow experience. Accordingly, twelve empirical studies were excluded because although the concept of flow was discussed, their data did not investigate flow experience. Forty-six articles were excluded because they were theoretical articles, reviews, meta-analyses or book chapters without original data. From the 257 publications, 199 empirical studies were included in the review (Table 1).

**TABLE 1:** Overview of the studies included in this review (*N* = 252).

Authors	Authors	Authors
Added based on the literature search	(*N* = 199)	

Asakawa and Csikszentmihalyi (2010) Bachen et al. (2016)* Bailis (2001) Bakker (2005)* Bakker et al. (2011)* Banfield and Burgess (2013) Bass (2007) Bassi and Delle Fave (2012a)* Bassi et al. (2014a) Bassi et al. (2014b)* Bassi et al. (2012)* Bassi et al. (2007) Baumann and Scheffer (2011)* Baumann et al. (2016)* Beard and Hoy (2010) Belchior et al. (2012)* Bonaiuto et al. (2016)* Boyns and Appelrouth (2011) Bressler and Bodzin (2013)* Bressler and Bodzin (2016)* Bricteux et al. (2017) Brinthaupt and Shin (2001) Brown and Westman (2008) Busch et al. (2013)* Byrne et al. (2003) Ceja and Navarro (2009) Chen and Lu (2016) Chen and Sun (2016) Chen et al. (2010) Cheok et al. (2011)* Coleman (2014) Csikszentmihalyi and Hunter (2003) Culbertson et al. (2015)* Dawoud et al. (2015)* Debus et al. (2014) Delespaul et al. (2004) Delle Fave and Bassi (2009)* Delle Fave and Massimini (2005)* Deol and Singh (2016) de Manzano et al. (2010) Demerouti (2006)* Diaz and Silveira (2013)* Drengner et al. (2008)* Eisenberger et al. (2005)* Emanuel et al. (2016) Engeser and Baumann (2016)* Engeser and Rheinberg (2008)* Escartin Solanelles et al. (2014) Faiola et al. (2013) Fink and Drake (2016) Freer (2009) Fullagar et al. (2013) Fullagar and Kelloway (2009)* Fulmer and Tulis (2016) Gaggioli et al. (2013)* Garces-Bacsal (2016) Gnoth et al. (2000) Graham (2008)*	Hudock (2015) Ivory and Magee (2009) Jackman et al. (2016)* Johnson et al. (2014) Jones (2013) Jonson et al. (2015)* Karageorghis et al. (2000)* Katuk et al. (2013) Kawabata and Mallett (2011) Kee and John Wang (2008) Keeler et al. (2015) Keller and Bless (2008)* Keller and Blomann (2008) Keller et al. (2011a)* Keller et al. (2011b)* Khan and Pearce (2015) Kim et al. (2014) Klasen et al. (2012)* Koehn and Morris (2014) Konradt and Sulz (2001)* Kopačević et al. (2011) Kuhnle et al. (2012) Kuhnle and Sinclair (2011) Lee (2013) Lee et al. (2016) Lee and LaRose (2007)* Liu and Shiue (2014) Liu et al. (2015)* Llorens et al. (2013) MacDonald et al. (2006)* MacNeill and Cavanagh (2013) Maeran and Cangiano (2013)* Magyaródi and Oláh (2015)* Mao et al. (2016) Marin and Bhattacharya (2013)* Marston (2013) Mesurado and Richaud de Minzi (2013) Mesurado et al. (2016)* Meyer et al. (2016)* Meyer and Jones (2013) Min et al. (2015)* Mirlohi et al. (2011) Montgomery et al. (2004) Moore (2013) Mosing et al. (2012) Moreno Murcia et al. (2008)* Nielsen and Cleal (2010) Nissen-Lie et al. (2015) Niu and Chang (2014)* Oertig et al. (2014)* Oertig et al. (2013) Ortner et al. (2014) Ozkara et al. (2016)* Páez et al. (2015) Panadero et al. (2014)* Panebianco-Warrens (2014)	Pratt et al. (2016) Rathunde (2010) Rathunde and Csikszentmihalyi (2005) Reynolds and Prior (2006)* Rha et al. (2005)* Robinson et al. (2012) Rodríguez-Sánchez et al. (2011a)* Rodríguez-Sánchez et al. (2011b)* Rogatko (2009) Ryu and Parsons (2012)* Salanova et al. (2014)* Sartori and Delle Fave (2014)* Sartori et al. (2014) Schattke (2011)* Schattke et al. (2014)* Schiefele and Raabe (2011) Schmierbach et al. (2014) Schmierbach et al. (2012) Schüler (2007)* Schüler and Brandstätter (2013) Schüler et al. (2010)* Schüler and Brunner (2009) Schüler and Nakamura (2013) Schüler et al. (2016)* Schweinle et al. (2008)* Seddon et al. (2008) Sharitt (2010) Shernoff et al. (2003)* Shin (2006)* Silverman et al. (2016) Sinnamon et al. (2012) Smith et al. (2012)* Steele and Fullagar (2009)* Stephanou (2011) Sugiyama and Inomata (2005)* Swann et al. (2017)* Swann et al. (2015a)* Swann et al. (2015b) Swann et al. (2012) Szymanski and Henning (2007) Tan and Chou (2011) Tanaka and Ishida (2015) Thin et al. (2011)* Thornton and Gilbert (2011) Tozman et al. (2015) Tramonte and Willms (2010) Tyagi et al. (2016) Ullén et al. (2012)* Ulrich et al. (2014)* Urmston and Hewison (2014)* van der Hoorn (2015) van Schaik et al. (2012)* Vealey and Perritt (2015) Harris et al. (2017) Valenzuela and Codina (2014)* Voiskounsky and Smyslova (2003) Vuorre and Metcalfe (2016) Wang and Hsu (2014)
Griffiths (2008) Guizzo and Cadinu (2016)* Guo and Poole (2009) Gute et al. (2008) Harris et al. (2017)* Hefferon and Ollis (2006) Heller et al. (2015)* Hernandez et al. (2014) Hong et al. (2013)* Hsu et al. (2013)*	Payne et al. (2011) Pearce et al. (2005)* Peifer et al. (2015) Peifer et al. (2014) Peterson and Miller (2004) Pilke (2004) Pinquart and Silbereisen (2010) Plester and Hutchison (2016) Pocnet et al. (2015)	Wang et al. (2015) Wanner et al. (2006) Winberg and Hedman (2008)* Wissmath et al. (2009)* Wrigley and Emmerson (2013)* Yan and Davison (2013) Zha et al. (2015) Zumeta et al. (2016)
Added by the experts:	(*N* = 41)	
Abuhamdeh and Csikszentmihalyi (2009) Armstrong (2008) Asakawa (2010) Aubé et al. (2014) Bassi and Delle Fave (2010) Berta et al. (2013) Butkovic et al. (2015) Coatsworth et al. (2005) Delle Fave et al. (2003) de Manzano et al. (2013) Demerouti et al. (2012)* Harmat et al. (2015) Heutte et al. (2016) Hirao and Kobayashi (2013) Hirao et al. (2012a)	Hirao et al. (2012b) Hodge et al. (2009) Inkinen et al. (2014) Jackson et al. (2001)* Kivikangas (2006) Kuo and Ho (2010) Collins et al. (2009) Mäkikangas et al. (2010) Martin and Cutler (2002) Meng et al. (2016) Mills and Fullagar (2008)* Moneta (2004) Montijo and Mouton (2016) Nacke and Lindley (2008)	Nacke et al. (2011) Novak et al. (2003) Salanova et al. (2006) Synofzik et al. (2008) Ullén et al. (2016) Ulrich et al. (2016a) Ulrich et al. (2016b) Walker (2010) Wolf et al. (2015) Yoshida et al. (2014) Zubair and Kamal (2015a) Zubair and Kamal (2015b)
Added from the EFRN publication list:	(*N* = 12)	
Bassi and Delle Fave (2012b) Baumann and Scheffer (2010)* Ceja and Navarro (2012) Ceja and Navarro (2011) Cseh et al. (2016)* Cseh et al. (2015)*	Moneta (2012)* Tobert and Moneta (2013) Vittersø (2003)* Vittersø et al. (2001) Voiskounsky et al. (2005) Wright et al. (2007)*	

*Marked articles were rated as fitting to more than one category.

### Charting the Information

During the 5th EFRN meeting in Milan (Italy), in November 2016, the preliminary framework of flow research as agreed during the 4th EFRN meeting was adapted. Based on the identified articles within our literature research, categories were added if necessary to adequately describe the literature. The final framework that was used in this Scoping Review is depicted in Figure 1.

During the meeting in Milan, experts from the EFRN were assigned to each category, and were responsible for that category in the following process. All experts are active flow researchers and members of the EFRN, who have published peer-reviewed papers in the field of their respective category. These experts are the team of authors of this Scoping Review.

In order to ensure a common understanding of the categories, the experts provided a clear description of their category. These were gathered, shared, and discussed between the authors. The outcome of step 4 was a final document which contained the agreed list of categories and their respective descriptions. This document forms the basis of the categorization of articles in the following step 5.

All articles were then distributed among the authors for them to rate their relevance for each category (see Figure 1) based on the abstracts. It was therefore possible that one article would be rated as being relevant for more than one category. Every article was independently reviewed by two authors. Empirical studies that were rated as relevant to the category by both authors were immediately included in the review of the category. Empirical studies that were only rated as relevant to the category by one author were again rated by the responsible expert(s). If he or she rated this article as relevant, it was also included in the review of the category. Otherwise, it was excluded.

### Collating, Summarizing and Reporting of Study Results

A large table listing all articles with their respective categories as rated by the authors was sent to the experts (i.e., the authors for a specific category) in order to start the process of summarizing the study results. In addition to the articles in the table, experts could include further empirical articles which had not been found in the initial search that they considered relevant for their respective category. That way, we aimed at providing a broad picture of flow research, as required in a Scoping Review. Forty-one additional empirical studies were included in the review by our experts and twelve articles from the EFRN publication list. Table 1 presents all included empirical studies. Next, experts extracted all relevant articles for their category from the large table and created a table of articles of their category. The final tables of included articles for each category can be found in the Results section for the respective categories.

Based on this extraction, and on the description of the category, experts summarized the results of articles placed in their assigned category, thereby ignoring findings reported in an article that did not belong to that category: 93 of the articles are represented in more than one category, each time with a different focus (see Table 1). To achieve a coherent manuscript without too many redundancies, the content of each category was revised during an internal review process.

### Discussion of the Results and Implications for Future Research

In addition to the summaries of the categories in the result section, experts collected points for discussion. These points were picked up and integrated into our general discussion of flow research, which built step 6 of our Scoping Review. During the 6th EFRN meeting in Tilburg (Netherlands, 2017), these points were discussed within the network and further elaborated. At this point, and in line with the aims of the EFRN, implications for future research which would foster scientific progress in flow research were identified.

## Results

The following section provides the expert summaries of each category. Table 2 provides an overview of all categories, the number of integrated articles and the operationalization of the respective category.

**TABLE 2:** Overview of categories.

Category	N	Studies were included, that…
Personality	40	… investigated personality traits and motives as stable individual factors. Furthermore, studies were included that dealt with heritability or genes of flow proneness and individual differences.
Motivation	54	… dealt with intrinsic or extrinsic motivation, interest and volition. Furthermore, studies were included that dealt with motivational concepts such as self-determination, self-efficacy, self-regulation and locus of control.
Physiology	21	… used physiological and/or neuropsychological methods (e.g., ECG, EEG, EMG, fMRI, eye-tracking, saliva sampling, etc.) to measure the relationship of physiological parameters with flow.
Emotion	49	… dealt with a wide range of concepts associated with different components of the emotional experience, which tends to be generally associated with a certain subjective degree of pleasure and displeasure, or positive and negative experiences, such as affect, mood, wellbeing, enjoyment, activation, or excitement.
Cognition	26	… dealt with perception, attention, decision-making and cognitive control. Also, brain studies referring to cognitive processes during flow experiences and effortless attention were included, as well as studies dealing with embodied cognition (e.g., body image, agency, intentions) and effects of flow experiences on cognitive processes (e.g., memory and reasoning).
Behavior	53	… dealt with flow and different forms of behavior such as performance (e.g., in-role/extra-role performance, physical, athletic, creative, or cognitive performance), risk taking, consumption behavior, online behavior and addiction, as well as variables that are closely related to performance and motivate high performance such as engagement, commitment, and persistence.
Context factors	94	… investigated different contexts and activities in which flow occurs (e.g., different kinds of work, study, sports etc.), as well as contextual characteristics/external circumstances that foster or hinder flow (e.g., differences in environmental characteristics, external demands and resources).
interindividual factors	13	… dealt with flow in social contexts, measured at the individual or collective level and as a social phenomenon (e.g., team flow, group flow, social flow etc.). Also, studies were included, which looked at the effects of flow on more than one individual (e.g., small groups, social settings, networks, and other collectives).
Cultural factors	16	… did cross-cultural investigations on flow. Furthermore, studies were included that dealt with individualism or collectivism, culture and the construction of the self, social identity, or special artifacts (e.g., Manga). Additionally, studies are included that addressed specific countries.

### Personality

The category *Personality and Flow* included studies that investigated personality traits and motives as stable individual factors. Studies that dealt with heritability or genes of flow proneness and individual differences were also included. Expert ratings revealed that 31 articles have met these inclusion criteria. Seven additional articles were included by our experts and two articles from the EFRN publication list. The final list of articles that were integrated into this section is depicted in Table 3.

**TABLE 3:** Personality.

Authors	Authors
Bailis (2001) Bassi et al. (2014b) Baumann and Scheffer (2011) Baumann et al. (2016) Beard and Hoy (2010) Busch et al. (2013) Fullagar and Kelloway (2009) Heller et al. (2015) Jackman et al. (2016) Johnson et al. (2014) Keller and Bless (2008) Keller and Blomann (2008) Kuhnle et al. (2012) Liu et al. (2015) Marin and Bhattacharya (2013) Mesurado and Richaud de Minzi (2013)	Mosing et al. (2012) Moreno Murcia et al. (2008) Oertig et al. (2014) Peterson and Miller (2004) Schattke (2011) Schattke et al. (2014) Schüler (2007) Schüler and Brandstätter (2013) Schüler et al. (2010) Schüler et al. (2016) Sinnamon et al. (2012) Sugiyama and Inomata (2005) Tan and Chou (2011) Ullén et al. (2012) Vealey and Perritt (2015)
Added by the experts:	
Abuhamdeh and Csikszentmihalyi (2009) Butkovic et al. (2015) de Manzano et al. (2013) Hirao et al. (2012b)	Mills and Fullagar (2008) Moneta (2004) Ullén et al. (2016)
Added from EFRN publication list:	
Baumann and Scheffer (2010) Vittersø (2003)	

*Studies were included that investigated personality traits and motives as stable individual factors. Furthermore, studies were included that dealt with heritability or genes of flow proneness and individual differences.*

The personality studies on flow can be divided into four categories: (1) studies dealing with autotelic personality, (2) dispositional proneness to experience flow and its relation to Big Five personality traits, (3) the relationship of flow with other personality traits or motives and (4) flow and motive-fitting situations.

#### Studies Dealing With Autotelic Personality

Autotelic personality is the ability to enter a flow state relatively easily (Csikszentmihalyi and Csikszentmihalyi, 1988) which was investigated in an interview-study from Sugiyama and Inomata (2005). Moneta (2004) and Abuhamdeh and Csikszentmihalyi (2009) state that intrinsic motivation is associated with autotelic personality, but little is known about its exact components. Existing studies suggest that these components of autotelic personality are personal innovativeness, self-efficacy, control, focused attention (Tan and Chou, 2011), and the achievement motive (Baumann and Scheffer, 2011; Busch et al., 2013).

#### Dispositional Proneness to Experience Flow and Its Relation to Big Five Personality Traits

Flow proneness is a dispositional tendency to experience flow and there are large individual differences in the frequency and intensity of flow experiences. Several self-report questionnaires have been developed to measure the variation between individuals in flow proneness e.g., Jackson and Eklund’s Dispositional Flow Scale-2 (Jackson and Eklund, 2002; Jackson et al., 2008; e.g., applied by Sinnamon et al., 2012, Johnson et al., 2014); and the Swedish Flow Proneness Questionnaire (SFPQ, Ullén et al., 2012). Existing studies suggest that flow proneness is related to well-established personality traits and that this association has a biological basis: Ullén et al. (2012) found that flow proneness is correlated with the Big Five personality traits emotional stability (i.e., low neuroticism) and conscientiousness. In addition, trait flow is related to extraversion, openness to experience, and agreeableness (Ullén et al., 2016). Other studies found that dispositional flow is associated with high extraversion and low neuroticism, and trait emotional intelligence in musicians (Marin and Bhattacharya, 2013; Heller et al., 2015). In addition, openness and music-specific flow were found to be the strongest predictors of music practice (Butkovic et al., 2015). In line with this, further studies suggest that extraversion and openness to experience are positively related to flow, while high neuroticism and introversion related to less flow experience (Vittersø, 2003; Baumann and Scheffer, 2010; Mesurado and Richaud de Minzi, 2013; Bassi et al., 2014b; Heller et al., 2015).

#### The Relationship of Flow With Other Personality Traits or Motives

Other personality traits also seem to be associated with flow experience: Bailis (2001) found that athletes’ trait self-handicapping score was positively related to optimal experience in competition. High mental toughness, i.e., a personal capacity supporting the process of high performance (Jackman et al., 2016), perceived motivational climates, and individuals’ goal orientations (Moreno Murcia et al., 2008) could account for differences in dispositional flow in athletes. Further, Kuhnle et al. (2012) found that self-control predicted flow experiences in eighth graders. Keller and Blomann (2008) found that a strong internal locus of control fosters flow under a skill-demand fit. Furthermore, studies suggest that action orientation fosters flow under skill-demand fit (Keller and Bless, 2008) and even under suboptimal (no skill-demand fit) conditions (Baumann et al., 2016). Beard and Hoy (2010; using state flow) and Vealey and Perritt (2015; using dispositional flow) found that optimism was positively related to flow whereas another study with Japanese students found that shyness predicted the frequency of flow experience (Hirao et al., 2012b). However, while empirical studies show that personality factors foster flow experiences, situational factors seem to have a bigger effect on flow (Fullagar and Kelloway, 2009; Ullén et al., 2016).

Using the SFPQ, Mosing et al. (2012) measured genetic influences on flow proneness in a cohort of adult twins and multivariate twin modeling indicated a moderate heritability of flow proneness. De Manzano et al. (2013) used positron emission tomography (PET) and found a positive relation between flow proneness and D2 receptor availability in the striatum. Their results suggested that the differences in the dopamine system could reflect personality differences.

#### Flow and Motive-Fitting Situations

Studies indicate that motives foster flow experiences in motive-fitting situations (Schattke, 2011; Oertig et al., 2014; Schüler et al., 2016). For example, Schüler et al. (2016) found that people scoring high on the autonomy motive experience flow in situations that satisfied participant’s autonomy-motive. Furthermore, Mills and Fullagar (2008) found that the need for autonomy moderated the relationship between flow and intrinsic motivation. Oertig et al. (2014) found that a high avoidance motive results in greater flow when performance-avoidance goals were induced. Schüler et al. (2010) found that the feeling of competence resulted in higher flow of participants who had a high achievement motive in sports [see also Schüler and Brandstätter (2013)]. Furthermore, high achievement motive and high hope of success were positively correlated with flow experience of wall climbers’ and students (Peterson and Miller, 2004; Schüler, 2007; Schattke, 2011; Schattke et al., 2014).

### Motivation

The category *Motivation and Flow* included studies that dealt with intrinsic or extrinsic motivation, interest, and volition. Also included were studies that dealt with motivational concepts such as self-determination, self-efficacy, self-regulation, and locus of control. Expert ratings revealed that 44 articles have met these inclusion criteria. Another eight articles were included by our experts and two articles from the EFRN publication list. The final list of articles that were integrated into this section is depicted in Table 4.

**TABLE 4:** Motivation.

Authors	Authors
Bachen et al. (2016) Bassi and Delle Fave (2012a) Baumann and Scheffer (2011) Belchior et al. (2012) Bonaiuto et al. (2016) Bressler and Bodzin (2013) Bricteux et al. (2017) Busch et al. (2013) Chen and Lu (2016) Chen and Sun (2016) Delle Fave and Massimini (2005) Eisenberger et al. (2005) Engeser and Rheinberg (2008) Fulmer and Tulis (2016) Gaggioli et al. (2013) Jackman et al. (2016) Karageorghis et al. (2000) Keller and Bless (2008) Keller et al. (2011a) Keller et al. (2011b) Kim et al. (2014) Lee and LaRose (2007)	Mesurado et al. (2016) Meyer et al. (2016) Montgomery et al. (2004) Moreno Murcia et al. (2008) Oertig et al. (2014) Oertig et al. (2013) Ozkara et al. (2016) Plester and Hutchison (2016) Pocnet et al. (2015) Rha et al. (2005) Rodríguez-Sánchez et al. (2011a) Rodríguez-Sánchez et al. (2011b) Salanova et al. (2014) Schattke (2011) Schattke et al. (2014) Schüler et al. (2016) Schüler et al. (2010) Shernoff et al. (2003) Steele and Fullagar (2009) Ulrich et al. (2014) Valenzuela and Codina (2014) Yan and Davison (2013)
Added by the experts:	
Armstrong (2008) Heutte et al. (2016) Hodge et al. (2009) Martin and Cutler (2002)	Mills and Fullagar (2008) Novak et al. (2003) Salanova et al. (2006) Walker (2010)
Added from the EFRN publication list:	
Bassi and Delle Fave (2012b) Moneta (2012)	

*Studies included dealt with intrinsic or extrinsic motivation, interest and volition. Furthermore, studies that dealt with motivational concepts such as self-determination, self-efficacy, self-regulation, and locus of control were included.*

The motivation studies on flow can be divided into four categories: studies dealing with flow and (1) motivational indicators (volition, engagement, goal orientation, achievement motive, interest, intrinsic motivation), (2) self-determination (3) self-efficacy, and (4) social motivation.

#### Motivational Indicators

If “motivation” can be simplistically defined as “move to action,” for its part, “volition” can be simplistically defined as “will to persist in action.” Thus, if motivation promotes an intention to act, then volition protects it (Corno, 2001). It was found that volition is positively linked to flow (e.g., Schattke, 2011). Another motivational indicator associated with flow is engagement, which “reflects an employee’s intention to throw their full self—heads, hands, and heart—into their work” (Plester and Hutchison, 2016, p. 4). Many studies investigated the association between the two concepts (e.g., Karageorghis et al., 2000; Shernoff et al., 2003; Montgomery et al., 2004; Rha et al., 2005; Steele and Fullagar, 2009; Belchior et al., 2012; Ulrich et al., 2014; Valenzuela and Codina, 2014; Pocnet et al., 2015; Mesurado et al., 2016; Plester and Hutchison, 2016). Goal orientation was also found to be linked to flow (e.g., Delle Fave and Massimini, 2005; Moreno Murcia et al., 2008; Schüler et al., 2010; Oertig et al., 2013, 2014; Bonaiuto et al., 2016; Jackman et al., 2016; Ozkara et al., 2016), as well as the achievement motive (e.g., Engeser and Rheinberg, 2008; Baumann and Scheffer, 2011; Busch et al., 2013; Schüler and Brandstätter, 2013; Schattke et al., 2014; see Personality and Flow). Furthermore, interest, which can be described as a motivational state resulting from attraction to a certain domain or activity (Reeve, 2008), was found to be related to flow (e.g., Eisenberger et al., 2005; Bressler and Bodzin, 2013; Bachen et al., 2016; Bricteux et al., 2017). Intrinsic motivation was investigated particularly often in its relation to flow, with evidence for a positive link found in various settings, such as education (Schüler et al., 2010; Keller et al., 2011b; Valenzuela and Codina, 2014; Meyer et al., 2016), Information and Communication Technologies (ICT) use (Voiskounsky and Smyslova, 2003; Montgomery et al., 2004; Keller and Bless, 2008; Yan and Davison, 2013; Kim et al., 2014; Chen and Lu, 2016); daily activities (Gaggioli et al., 2013) and physiological aspects (Keller et al., 2011a; Ulrich et al., 2014).

#### Self-Determination

Self-determination theory (SDT) “is an empirically derived theory of human motivation and personality in social contexts that differentiates motivation in terms of being autonomous and controlled” (Deci and Ryan, 2012, p. 416). Autonomous motivation combines forms of intrinsic motivation with those forms of extrinsic motivation, which go along with a sense of identification with the activity and its values; accordingly, it goes along with increased volition and self-endorsement (Deci and Ryan, 2008). In contrast, controlled motivation is associated with experiencing the “pressure to think, feel, or behave in particular ways” (Deci and Ryan, 2008, p. 182). Many authors (e.g., Schüler et al., 2010; Schattke, 2011; Bassi and Delle Fave, 2012a,b; Fulmer and Tulis, 2016) consider that flow experience is linked to autonomous motivation. Studies which examine flow in the context of self-determination theory showed for example that work-related flow is associated with both autonomous regulation and controlled regulation (Bassi and Delle Fave, 2012a). Furthermore, raising children in a way that promotes self-determination will help them to engage in activities which will enhance their flow experience (Schattke, 2011). In another study, it was found that flow enhanced learning motivation in computer-based learning systems if participants experienced self-control (Kim et al., 2014). Goal-directed activities with clear instructions are supported in environments where the individual feels autonomous and self-determined (e.g., providing choices). These activities are motivating as well as flow-inducing (Novak et al., 2003). Conceptually, and on the approach-avoidance spectrum, the approach aspect of goals is likely to promote intrinsic motivation because it facilitates challenge appraisals and task absorption, whereas the avoidance aspect of goals is likely to undermine intrinsic motivation because it evokes threat appraisals, anxiety, and self-concern (Elliot, 2005).

#### Self-Efficacy

This category of studies within this section reviews studies dealing with flow and self-efficacy, i.e. the “people’s judgments of how well they can organize and execute, constituent cognitive, social, and behavioral skills in dealing with prospective situations” (Bandura, 1983, p. 467). The degree of self-efficacy affects the initiation, persistence and effort in activities (Bandura, 1977), and is, thus, an influential motivational theory. Results of empirical studies confirm that self-efficacy is linked with flow frequency and higher levels of challenge and skills showing that self-efficacy predicts flow over time (Rodríguez-Sánchez et al., 2011a; Heutte et al., 2016). Collective efficacy beliefs predict collective flow over time (Salanova et al., 2014, see sections *Interindividual Factors and Flow and Cognition and Flow*). High levels of efficacy beliefs have a positive impact on flow experiences in academic settings (Salanova et al., 2006; Bassi et al., 2007; Heutte et al., 2016). Various aspects of Bandura’s (1986) self-regulation learning model were shown to exert a significant and positive effect on flow (Lee and LaRose, 2007; Rodríguez-Sánchez et al., 2011a; Chen and Sun, 2016).

#### Social Motivation

Some first studies highlight the social motivational conditions of flow (Sawyer, 2003; Armstrong, 2008; Walker, 2010; Heutte et al., 2016). Although this requires further investigation, it seems that the quality of interpersonal relationships, supporting in particular basic psychological needs (autonomy, competence, and relatedness), will support a motivational climate favorable to the emergence of flow within a group.

### Physiology

The category *Physiology and Flow* included studies that used physiological and/or neuropsychological methods (e.g., ECG, EEG, EMG, fMRI, eye-tracking, saliva sampling, etc.) to measure the relationship of physiological parameters with flow. Expert ratings revealed that nine articles meet these inclusion criteria. Another twelve articles were included by the experts. The final list of articles integrated into this section is set out in Table 5.

**TABLE 5:** Physiology.

Authors	
de Manzano et al. (2010) Gaggioli et al. (2013) Harris et al. (2017) Keller et al. (2011a) Klasen et al. (2012)	Peifer et al. (2015) Peifer et al. (2014) Tozman et al. (2015) Ulrich et al. (2014)
Added by the experts:	
Berta et al. (2013) Brom et al. (2014) Harmat et al. (2015) Hirao et al. (2012a) Kivikangas (2006) Meng et al. (2016)	Nacke and Lindley (2008) Nacke et al. (2011) Ulrich et al. (2016a) Ulrich et al. (2016b) Wolf et al. (2015) Yoshida et al. (2014)

*Studies included used physiological and/or neuropsychological methods (e.g., ECG, EEG, EMG, fMRI, eye-tracking, saliva sampling, etc.) to measure the relationship of physiological parameters with flow.*

Subtopics identified in the literature include flow’s relationship with (1) physiological arousal as represented by sympathetic (SA) and parasympathetic activation (PA), and cortisol, (2) facial muscle activation (FMA) and (3) neural activity.

#### Physiological Arousal

Flow was found to relate negatively to cardiac output and systolic blood pressure, and positively to diastolic blood pressure and heart rate (de Manzano et al., 2010; Gaggioli et al., 2013; Harris et al., 2017). Furthermore, mixed associations of flow with SA were found, with some studies showing positive associations (Nacke and Lindley, 2008; de Manzano et al., 2010; Gaggioli et al., 2013; Ulrich et al., 2016b), other studies showing negative associations (Harmat et al., 2015; Tozman et al., 2015; Harris et al., 2017) and—under stress—the relationship was found to be inverted u-shaped (Peifer et al., 2014; Tozman et al., 2015). Two studies found no association between flow and SA (Kivikangas, 2006; Hirao et al., 2012a). Similarly, PA has been negatively associated with flow (de Manzano et al., 2010; Keller et al., 2011a), but under stress, studies identified a positive relationship (Peifer et al., 2014) and an inverted u-shaped relationship (Tozman et al., 2015). Respiratory depth, related to PA, increased during flow (de Manzano et al., 2010). Regarding flow and cortisol, studies have found a positive association (Keller et al., 2011a), no association (Brom et al., 2014), a negative effect of high cortisol on flow (Peifer et al., 2015) and an inverted u-shaped relationship between cortisol and flow in stress-relevant conditions (Peifer et al., 2014; Tozman et al., 2015).

#### Facial Muscle Activation

Studies examining FMA found associations with flow for the Zygomaticus Major (de Manzano et al., 2010; Nacke et al., 2011), Orbicularis Oculi (Nacke et al., 2011), and Corrugator Supercilii (Kivikangas, 2006). In this sub-category, findings were also inconsistent.

#### Neural Activity

Neuroscientific research showed that flow was characterized by greater activation of the “multiple-demand system,” which is involved in task-relevant cognitive functions, and reduced activation of the default mode network (*via* a relative increase in the dorsal raphe nucleus), which is linked to self-referential processing (Ulrich et al., 2014, 2016a,2016b). Computer gamers reporting flow showed increased activity in the neocerebellum, somatosensory cortex, and motor areas, possibly indicating a synchronization between reward-related brain structures and task-relevant cortical and cerebellar areas during flow (Klasen et al., 2012). Larger stimulus-preceding negativities (SPNs) were found during flow, indicating increased motivation and anticipatory attention (Meng et al., 2016). Experts experiencing more flow had greater right temporal cortical activity when imagining the activity, possibly reflecting the automaticity of a highly trained skill (Wolf et al., 2015).

Of particular interest is frontal activity during flow, inspired by the Hypofrontality Hypothesis suggested by Dietrich (2004). The Hypofrontality Hypothesis states that analytical and meta-conscious capacities are temporarily suppressed during flow, physiologically indicated by a downregulation of prefrontal activity. Respective findings support no association of flow with frontal activity (Harmat et al., 2015), or a greater activation of the ventrolateral prefrontal cortex (Yoshida et al., 2014). Findings regarding EEG activity were similarly mixed: Nacke et al. (2011) found no relationship, while Berta et al. (2013) found that alpha and lower- and mid-beta power predicted flow.

### Emotion

The category *Emotion and Flow* included studies that dealt with a wide range of concepts associated with different components of the emotional experience, which tends to be generally associated with a certain subjective degree of pleasure and displeasure, or positive and negative experiences, such as affect, mood, wellbeing, enjoyment, activation, or excitement. Although a unique and clear definition of emotion does not exist in these articles, the relation of emotion with flow experience seems to follow a clear understanding of the kind of emotional components that can be relevant when studying this relationship. Although the concept of emotion, in its broad sense, can integrate cognitive, affective, and behavioral or even physiological aspects, this section tried to avoid overlapping with others that are specifically devoted to one of these components in its relation with flow experience (e.g., cognition and flow). Expert ratings revealed that 40 articles have met these inclusion criteria. Four additional articles were included by our experts and five articles from the EFRN publication list. The final list of articles that were integrated into this section is depicted in Table 6.

**TABLE 6:** Emotion.

Authors	Authors
Bachen et al. (2016) Bassi et al. (2014a) Bassi et al. (2014b) Chen et al. (2010) Culbertson et al. (2015) Csikszentmihalyi and Hunter (2003) Delespaul et al. (2004) Delle Fave and Massimini (2005) Diaz and Silveira (2013) Eisenberger et al. (2005) Engeser and Baumann (2016) Fink and Drake (2016) Fullagar et al. (2013) Fullagar and Kelloway (2009) Graham (2008) Hsu et al. (2013) Karageorghis et al. (2000) Kopačević et al. (2011) Maeran and Cangiano (2013) Marin and Bhattacharya (2013)	Ozkara et al. (2016) Páez et al. (2015) Panadero et al. (2014) Pinquart and Silbereisen (2010) Rathunde (2010) Robinson et al. (2012) Rogatko (2009) Sartori and Delle Fave (2014) Schmierbach et al. (2014) Schüler (2007) Schweinle et al. (2008) Shin (2006) Silverman et al. (2016) Steele and Fullagar (2009) Sugiyama and Inomata (2005) Thin et al. (2011) Tramonte and Willms (2010) Tyagi et al. (2016) Wanner et al. (2006) Wissmath et al. (2009)
Added by the experts:	
Demerouti et al. (2012) Hirao and Kobayashi (2013)	Inkinen et al. (2014) Collins et al. (2009)
Added from the EFRN publication list:	
Baumann and Scheffer (2010) Cseh et al. (2015) Tobert and Moneta (2013)	Vittersø (2003) Wright et al. (2007)

*Studies included dealt with a wide range of concepts associated with different components of the emotional experience, which tends to be generally associated with a certain subjective degree of pleasure and displeasure, or positive and negative experiences, such as affect, mood, wellbeing, enjoyment, activation, or excitement.*

The identified studies show four main subtopics, i.e., (1) affect, (2) wellbeing, (3) enjoyment, and (4) emotional contagion. Studies investigated relationships of the emotional concepts with several components of flow, in particular with challenge-skill balance (Delespaul et al., 2004; Delle Fave and Massimini, 2005; Sugiyama and Inomata, 2005; Schweinle et al., 2008; Tramonte and Willms, 2010; Robinson et al., 2012; Panadero et al., 2014; Sartori and Delle Fave, 2014). In general, these studies showed that high challenge-skill balance is associated with higher positive emotional states (e.g., activation, excitement, positive affect).

#### Affect

Regarding the first subtopic, several studies suggest a positive relationship between flow and positive affect. Of relevance is the study by Baumann and Scheffer (2010) showing that achievement flow is supported by dynamic changes in positive affect, highlighting the role of reduced and restored positive affect. Some other findings show that flow predicts positive mood or positive affect (Eisenberger et al., 2005; Schüler, 2007; Collins et al., 2009; Fullagar and Kelloway, 2009; Baumann and Scheffer, 2010; Tobert and Moneta, 2013; Inkinen et al., 2014; Bachen et al., 2016; Ozkara et al., 2016). The reverse relationship also exists, with studies demonstrating that both positive and negative affect are significant predictors of flow experience (e.g., Collins et al., 2009; Kopačević et al., 2011; Hirao and Kobayashi, 2013; Tobert and Moneta, 2013). Cseh et al. (2015) demonstrated that flow is purported to have positive consequences on affect and performance. Some other studies looked at the relationship between flow and affect in different groups of participants (Rogatko, 2009; Fullagar et al., 2013; Bassi et al., 2014a; Fink and Drake, 2016; Tyagi et al., 2016), different activities or domains (Pinquart and Silbereisen, 2010; Engeser and Baumann, 2016; Silverman et al., 2016), or in relation to specific variables, for example, the quality of a relationship or experiential wisdom (Karageorghis et al., 2000; Graham, 2008; Rathunde, 2010), and trait emotional intelligence (Marin and Bhattacharya, 2013; see *Personality and Flow*).

#### Wellbeing

In studies considering wellbeing, flow experience tends to be positively associated with the concept of emotional wellbeing (Wanner et al., 2006), and psychological wellbeing (Bassi et al., 2014a,b), with others showing that flow experience can predict psychological wellbeing (Steele and Fullagar, 2009; Bassi et al., 2014b), life satisfaction (Collins et al., 2009; Chen et al., 2010; Bassi et al., 2014b), happiness (Csikszentmihalyi and Hunter, 2003), job satisfaction (Maeran and Cangiano, 2013), course satisfaction (Shin, 2006), and e-satisfaction and e-loyalty (Hsu et al., 2013).

#### Enjoyment

Regarding enjoyment, studies showed that it is positively associated with flow, with authors trying to understand which flow dimensions are related to the perception of enjoyment and under what circumstances (Wright et al., 2007; Wissmath et al., 2009; Thin et al., 2011; Diaz and Silveira, 2013; Inkinen et al., 2014; Schmierbach et al., 2014). In a diary study which aimed at examining the relationship between flow experiences and energy both during work and non-work, results indicated that the flow-characteristics absorption and enjoyment were associated with energy only after work, accompanied by feeling more vigorous and less exhausted (Demerouti et al., 2012).

#### Emotional Contagion

Two studies brought the topic of flow to collective and group contexts. It was shown that positive collective gatherings could stimulate shared flow experiences, promoting personal wellbeing and social cohesion (Zumeta et al., 2016). In the group context of a classroom, it was shown that Students’ perceptions of their classmates’ flow as well as their teachers’ flow were related to their own reported flow experience (Culbertson et al., 2015). Authors concluded that their finding can be explained by contagion effects of flow within the group, in line with emotional contagion theory (Hatfield et al., 1994).

### Cognition

The category *Cognition and Flow* included studies that dealt with perception, attention, decision-making, and cognitive control. Also, brain studies referring to cognitive processes during flow experiences and effortless attention were reviewed in this section. Studies dealing with embodied cognition (e.g., body image, agency, intentions) and effects of flow experiences on cognitive processes (e.g., memory and reasoning) were reviewed. Expert ratings revealed that 26 articles met these inclusion criteria. Two additional articles were included by our experts and one article from the EFRN publication list. The final list of articles that were integrated into this section is presented in Table 7.

**TABLE 7:** Cognition.

Authors	Authors
Delle Fave and Massimini (2005) Diaz and Silveira (2013) Guizzo and Cadinu (2016) Harris et al. (2017) Kawabata and Mallett (2011) Kee and John Wang (2008) Klasen et al. (2012) Konradt and Sulz (2001) Kuhnle and Sinclair (2011) Lee and LaRose (2007) Moore (2013) Ortner et al. (2014) Ozkara et al. (2016)	Payne et al. (2011) Pearce et al. (2005) Reynolds and Prior (2006) Rodríguez-Sánchez et al. (2011a) Schiefele and Raabe (2011) Schweinle et al. (2008) Swann et al. (2017) Swann et al. (2015a) Ullén et al. (2012) Ulrich et al. (2014) Vuorre and Metcalfe (2016) Winberg and Hedman (2008) Wissmath et al. (2009)
Added by the experts:	
Jackson et al. (2001) Synofzik et al. (2008)	
Added from the EFRN publication list:	
Cseh et al. (2016)	

*Studies included dealt with perception, attention, decision-making and cognitive control. Also, brain studies referring to cognitive processes during flow experiences and effortless attention were included, as well as studies dealing with embodied cognition (e.g., body image, agency, intentions) and effects of flow experiences on cognitive processes (e.g., memory and reasoning).*

Cognition studies on flow can be divided into two main areas: (1) those that studied its relationships with cognitive processes, and (2) those that analyzed cognitive aspects of flow-related processes while considering flow in specific applied contexts.

First of all, flow itself can be considered a state of consciousness in which an individual is fully concentrated on, paying attention to and engaged in a certain activity (Delle Fave and Massimini, 2005); at the same time, flow can be considered as a process or a dynamic mental activity characterized by clear goals, a match between capacity and challenge, absence of disturbances, experience of mastery, etc. (Pearce et al., 2005; Kawabata and Mallett, 2011). There is not a discrepancy between state and process—rather they can be seen as related and interdependent; a flow state typically occurs when an individual engages in a process with the formerly mentioned characteristics.

#### Relationships With Cognitive Processes

Flow is related to attentional processes. For example, as demonstrated by Harris et al. (2017), sustained attention toward the task is needed as a component of flow. Indeed, from a cognitive point of view, when attention is hindered by other processes or stimuli, flow experience is reduced or blocked. For instance, in the experiment by Guizzo and Cadinu (2016), feeling objectified by men’s gaze draws women’s attention away from the rewarding activity and decreases flow. However, studies on flow proneness highlight no relation or very weak relation with intelligence in two large samples (Ullén et al., 2012), showing that although flow is related to cognitive processes, it is only weakly associated with cognitive ability. In general, cognitive studies tend to confirm the skill-demands compatibility hypothesis in the generation of flow (Payne et al., 2011; Schiefele and Raabe, 2011; Harris et al., 2017). Moreover, flow has been found to be positively related to an intuitive approach to decision making (Kuhnle and Sinclair, 2011). Consistently, flow seems to be disassociated from sense of agency or the impression of being the author of one’s own actions (Vuorre and Metcalfe, 2016). Indeed, sense of agency is partially influenced by metacognitive, complex judgments of authorship over the action (Synofzik et al., 2008), which are more influenced by overall evaluation of one’s own control over the task, while flow appears to be associated with positive assessment and enjoyment of the overall experience. In other words, the reporting of having experienced an optimal experience is not related to feel more or less to be the author of one’s own actions. Neuropsychological data also showed that flow is associated with sense of control (Ulrich et al., 2014, see *Physiology and Flow*). Further, it was found that cognitive flexibility (Moore, 2013) and mindfulness predicted flow (Kee and John Wang, 2008; Moore, 2013). Studies on flow involving creative activities highlighted that flow was not affected by cognitive load (Cseh et al., 2016). Rather, flow experience could help banish or reduce unwanted cognitive processes (e.g., intrusive thoughts, rumination), for example in cancer patients (Reynolds and Prior, 2006).

#### Cognitive Aspects of Flow-Related Processes in Specific Contexts

The most popular field of research with regards to flow and cognitive processes are studies related to learning. Ortner et al. (2014) analyzed the effects of computerized adaptive testing (CAT) vs. computerized fixed item testing (FIT) on Students’ motivation and flow using a matrices non-verbal computer-based test assessing reasoning on the basis of figural items. The CAT version adapts to the learner’s online performance selecting items on the basis of the learner’s previous response, while the FIT version features fixed items increasing in difficulty. Contrary to hypotheses, fixed item testing obtained superior ratings of motivation and no differences between the conditions were found for flow. In a study by Konradt and Sulz (2001), most of the participants entered flow while using a hypermedia learning system, independently of task condition (scanning or browsing the contents); importantly, however, flow was not associated with improved learning. Diaz and Silveira (2013) analyzed flow experiences in high school music students attending a summer music camp; the highest ranked flow-inducing activities showed strong associations between attention and enjoyment. Another study (Winberg and Hedman, 2008) compared guiding/open instructions during a learning task and considered their effects on flow components. Guiding instructions correlated with high levels of “challenge,” “enjoyment,” and “concentration” and low levels of “perception of control,” while the opposite happened for the other condition. However, Pearce et al. (2005) found that a “process” (rather than a state) model of flow more adequately explains students’ outcomes, in that skills may change over time during learning (e.g., growing). In this sense, flow should probably be measured more times than just once after or during the learning process. Schweinle et al. (2008) employed experience sampling methods to analyze flow following 12 class lessons. They found that individual affect was influenced by the interaction of challenge and skill while social affect and efficacy were more impacted by perceived skill than by challenge (see *Emotion and Flow*). This is consistent with studies attempting to integrate flow with social-cognitive theory, namely, the idea of behavior resulting from cognitive processes and external/environmental influences (Lee and LaRose, 2007; Rodríguez-Sánchez et al., 2011a). These studies found that high self-efficacy, or the belief about one’s own abilities to perform a given action, may be a predictor of optimal experience (see *Motivation and Flow*).

Another important field of flow research is sports. For example, Swann et al. (2017) employed interviews to explore the characteristics of clutch performances (i.e., performance under pressure) in professional athletes. They found that clutch performances are different from flow, in that they are characterized by heightened awareness, deliberate concentration and intense effort. Also, an “inductive” qualitative research study on golfers (Swann et al., 2015a), or in other words, a methodology that did not intend to confirm flow characteristics as described by traditional theory but instead intended to capture the experience of the participants as described by them, suggested that flow was self-aware, observable and characterized by altered cognitive and kinesthetic perceptions.

Finally, flow has been found to be positively related to transportation and spatial presence while watching movies (Wissmath et al., 2009). Transportation has been defined as the “process where all mental systems and capacities become focused on events in the narrative” (Green and Brock, 2000, p. 701), with high involvement and absorption of the user in the movie he or she is watching, while sense of presence consists in the sensation of “being” inside a real or virtual environment, related to the impression of being able to enact one’s own intentions (Triberti and Riva, 2015).

### Behavior

The category *Behavior and Flow* included studies that dealt with flow and different forms of behavior such as performance (e.g., in-role/extra-role performance, physical, athletic, creative, or cognitive performance), risk taking, consumption behavior, online behavior, and addiction, as well as variables that are closely related to performance and motivate high performance such as engagement, commitment, and persistence. Expert ratings revealed that 46 articles have met these inclusion criteria. Another six articles were included by our experts and one from the ERFN publication list, although they were not found in the literature search. The final list of articles that were integrated into this section is set out in Table 8.

**TABLE 8:** Behavior.

Authors	
Bakker et al. (2011) Bassi et al. (2012) Bassi et al. (2007) Baumann and Scheffer (2011) Bressler and Bodzin (2016) Brinthaupt and Shin (2001) Busch et al. (2013) Byrne et al. (2003) Cheok et al. (2011) Culbertson et al. (2015) Dawoud et al. (2015) Demerouti (2006) Drengner et al. (2008) Eisenberger et al. (2005) Engeser and Rheinberg (2008) Graham (2008) Griffiths (2008) Guizzo and Cadinu (2016) Harris et al. (2017) Heller et al. (2015) Hong et al. (2013) Hsu et al. (2013) Keller et al. (2011b) Konradt and Sulz (2001)	Liu and Shiue (2014) MacDonald et al. (2006) Marin and Bhattacharya (2013) Mesurado et al. (2016) Min et al. (2015) Niu and Chang (2014) Pearce et al. (2005) Pratt et al. (2016) Schüler (2007) Schüler and Brunner (2009) Schüler and Nakamura (2013) Schüler et al. (2010) Seddon et al. (2008) Shernoff et al. (2003) Swann et al. (2017) Swann et al. (2015a) Swann et al. (2015b) Szymanski and Henning (2007) Thornton and Gilbert (2011) Urmston and Hewison (2014) Valenzuela and Codina (2014) Wrigley and Emmerson (2013)
Added by the experts:	
Aubé et al. (2014) Delle Fave et al. (2003) Jackson et al. (2001)	Kuo and Ho (2010) Zubair and Kamal (2015a) Zubair and Kamal (2015b)
Added from the EFRN publication list:	
Cseh et al. (2015)	

*Studies included dealt with flow and different forms of behavior such as performance (e.g., in-role/extra-role performance, physical, athletic, creative, or cognitive performance), risk taking, consumption behavior, online behavior and addiction, as well as variables that are closely related to performance and motivate high performance such as engagement, commitment, and persistence.*

Within this category, the following subtopics could be identified: (1) The relationship between flow and different kinds of performance in different contexts, (2) variables that are related to high performance such as engagement and commitment, and (3) other forms of behavior such as risk taking, consumption behavior, online behavior, and addiction.

#### Performance

Most studies dealing with flow and behavior address the topic of performance, and they show a positive relationship between flow and performance in most cases (e.g., Demerouti, 2006; Engeser and Rheinberg, 2008; Min et al., 2015: productivity in design process). For work-related performance, it was found that flow at work is positively related with in-role (Demerouti, 2006) and extra-role performance (Eisenberger et al., 2005; Demerouti, 2006). Baumann and Scheffer (2011) additionally found that the flow achievement motive is positively associated with work efficiency according to multisource feedback. The positive effects of flow on performance could also be shown at the team-level (Aubé et al., 2014). Likewise, Kuo and Ho (2010) found that flow has positive effects on employee-reliability and paying attention to customers’ needs.

Besides work-related performance, several other studies deal with the topic of flow and athletic and physical performance (e.g., Bailis, 2001; Jackson et al., 2001): Most studies find a positive relation between flow and physical performance (Schüler and Brunner, 2009; Bakker et al., 2011), including performance under pressure (Swann et al., 2017). Similarly, training and preparation appear to have a positive effect on flow and mediate effects on performance (Schüler and Brunner, 2009; Swann et al., 2015b). Swann et al. (2015a) also find that flow is related to changes in the behavior of golfers (such as playing faster, staying calm, and showing a confident body language).

In terms of performance at school and/or cognitive performance in general, flow was found to be positively related to exam performance (Schüler, 2007), cognitive performance (Engeser and Rheinberg, 2008; Harris et al., 2017) and goal progress (Schüler et al., 2010). The achievement flow motive also predicts academic success (Busch et al., 2013). Guizzo and Cadinu (2016) find that low levels of flow are associated with decreased cognitive performance in an attention to response task. Furthermore, studies suggest that practice and learning in general are positively related to flow experience (Brinthaupt and Shin, 2001; Pearce et al., 2005; Marin and Bhattacharya, 2013; Valenzuela and Codina, 2014; Heller et al., 2015; Bressler and Bodzin, 2016) and that flow is positively associated with reengagement in a task (Keller et al., 2011b; Pratt et al., 2016). Another study found that flow and learning retention in gaming were also positively associated (Hong et al., 2013). Flow also presented positive effects on performance in online games (Thornton and Gilbert, 2011). Overall, there seems to be a positive relation between flow and enhanced performance (for an overview see Landhäußer and Keller, 2012). However, two studies did not find a positive association between flow and performance (Konradt and Sulz, 2001; Culbertson et al., 2015). The former authors, however, suggest that the students in their investigation experienced flow and therefore felt self-confident and were not open to learn for a following quiz (for more explanations, see Culbertson et al., 2015). Several studies find a positive relationship between flow experiences and enhanced creativity or engagement in creative tasks (Byrne et al., 2003; Griffiths, 2008; Cseh et al., 2015; Dawoud et al., 2015; Zubair and Kamal, 2015a,b), especially in the field of music (MacDonald et al., 2006; Wrigley and Emmerson, 2013).

#### Variables That Are Related to High Performance

With respect to variables that are related to high performance, flow seems to be positively related with student engagement in the classroom (Shernoff et al., 2003; Mesurado et al., 2016) and with learning engagement (Bassi et al., 2007). Furthermore, several studies have found a positive relation between the fact of “being active” and flow (Bassi et al., 2012: engagement in meaningful rehabilitation activities; Drengner et al., 2008; Graham, 2008; Dawoud et al., 2015). Another study by Seddon et al. (2008) finds while investigating a 6-year online collaboration (working together in an online setting) that flow and engagement in that collaboration were positively related.

#### Other Forms of Behavior

With respect to other forms of behavior, Schüler and Nakamura (2013) found that risk behavior and flow were positively associated but only for inexperienced climbers; the relationship is mediated by self-efficacy beliefs. In line with that, Delle Fave et al. (2003) found that the opportunity to experience flow motivates climbers to take part in a risky expedition. Urmston and Hewison (2014) also find a positive relationship between flow and risk taking in learning. A study by Szymanski and Henning (2007) found that flow was negatively related to women’s self-objectification behavior. Further studies on self-objectification behavior were not found. Furthermore, Niu and Chang (2014) found that flow is positively associated with unplanned buying and that it moderates the positive relationship between internet addiction and consumer behavior. Liu and Shiue (2014) found that flow fosters purchase intention in online games. Another study found that experiencing flow was positively related with engagement in a human-animal-interaction game (Cheok et al., 2011). Hsu et al. (2013) find that flow and e-loyalty are positively related.

### Context Factors

The category *Context Factors and Flow* included studies that investigated different contexts and activities in which flow occurs (e.g., different kinds of work, study, sports etc.), as well as contextual characteristics/external circumstances that foster or hinder flow (e.g., differences in environmental characteristics, external demands and resources). Expert ratings revealed that 84 articles met these inclusion criteria. Another three articles were included by our experts and seven from the ERFN publication list, although they were not found in the literature search. The final list of articles that were integrated into this section is shown in Table 9.

**TABLE 9:** Context factors.

Authors	Authors
Bakker (2005) Bakker et al. (2011) Banfield and Burgess (2013) Bass (2007) Bassi et al. (2012) Bassi and Delle Fave (2012a) Baumann et al. (2016) Belchior et al. (2012) Bonaiuto et al. (2016) Bressler and Bodzin (2013) Bressler and Bodzin (2016) Ceja and Navarro (2009) Cheok et al. (2011) Coleman (2014) Dawoud et al. (2015) Debus et al. (2014) Delle Fave and Bassi (2009) Demerouti (2006) Deol and Singh (2016) Diaz and Silveira (2013) Eisenberger et al. (2005) Emanuel et al. (2016) Engeser and Baumann (2016) Engeser and Rheinberg (2008) Escartin Solanelles et al. (2014) Faiola et al. (2013) Freer (2009) Gnoth et al. (2000) Guo and Poole (2009) Hefferon and Ollis (2006) Hernandez et al. (2014) Hong et al. (2013) Hsu et al. (2013) Hudock (2015) Ivory and Magee (2009) Jones (2013) Jonson et al. (2015) Katuk et al. (2013) Keller and Bless (2008) Khan and Pearce (2015) Koehn and Morris (2014) Lee (2013)	Llorens et al. (2013) MacNeill and Cavanagh (2013) Maeran and Cangiano (2013) Magyaródi and Oláh (2015) Marston (2013) Meyer et al. (2016) Meyer and Jones (2013) Min et al. (2015) Mirlohi et al. (2011) Nielsen and Cleal (2010) Nissen-Lie et al. (2015) Panadero et al. (2014) Panebianco-Warrens (2014) Peterson and Miller (2004) Pilke (2004) Rathunde and Csikszentmihalyi (2005) Reynolds and Prior (2006) Rha et al. (2005) Rodríguez-Sánchez et al. (2011b) Ryu and Parsons (2012) Sartori and Delle Fave (2014) Sartori et al. (2014) Schmierbach et al. (2012) Sharitt (2010) Shernoff et al. (2003) Shin (2006) Smith et al. (2012) Steele and Fullagar (2009) Stephanou (2011) Swann et al. (2015a) Swann et al. (2012) Thin et al. (2011) Urmston and Hewison (2014) van der Hoorn (2015) van Schaik et al. (2012) Harris et al. (2017) Voiskounsky and Smyslova (2003) Wang and Hsu (2014) Wang et al. (2015) Winberg and Hedman (2008) Wrigley and Emmerson (2013) Zha et al. (2015)
Added by the experts:	
Bassi and Delle Fave (2010) Demerouti et al. (2012) Mäkikangas et al. (2010)	
Added from the EFRN publication list:	
Ceja and Navarro (2012) Ceja and Navarro (2011) Cseh et al. (2016) Moneta (2012)	Vittersø et al. (2001) Voiskounsky et al. (2005) Wright et al. (2007)

*Studies included investigated different contexts and activities in which flow occurs (e.g., different kinds of work, study, sports etc.), as well as contextual characteristics/external circumstances that foster or hinder flow (e.g., differences in environmental characteristics, external demands and resources).*

In this category, the following subtopics were identified: (1) Flow in different contexts and activities and how they affect flow, (2) contextual factors and their relationships with flow, and (3) the fit of contextual factors with characteristics of the individual.

#### Flow in Different Contexts and Activities

Flow is always investigated during a certain activity in a certain context, and their variety in the identified studies is large: (a) work- or study-related activities such as work, learning (Peterson and Miller, 2004; Rathunde and Csikszentmihalyi, 2005; Wright et al., 2007; Ceja and Navarro, 2011; Stephanou, 2011; Demerouti et al., 2012; Ryu and Parsons, 2012; Debus et al., 2014; Escartin Solanelles et al., 2014; Hernandez et al., 2014), and teaching (Coleman, 2014), (b) leisure (Rodríguez-Sánchez et al., 2011b), (c) professional dancing (Hefferon and Ollis, 2006; Panebianco-Warrens, 2014), (d) music festivals (Jonson et al., 2015), (e) creative activities such as designing clothes (Min et al., 2015) and visiting arts courses or making art (Reynolds and Prior, 2006; Bass, 2007; Jones, 2013; van der Hoorn, 2015), (f) gaming (e.g., Ivory and Magee, 2009; Thin et al., 2011; Bressler and Bodzin, 2013, 2016) and several online activities (e.g., Guo and Poole, 2009; Faiola et al., 2013; Hsu et al., 2013; Meyer and Jones, 2013; Wang et al., 2015), (g) research activities (Hudock, 2015; Zha et al., 2015) and information technology use (Pilke, 2004), (h) sports (e.g., Koehn and Morris, 2014; Deol and Singh, 2016; training vs. competition; Swann et al., 2012, 2015a), (i) translation activities (Mirlohi et al., 2011), (j) psychological rehabilitation activities (e.g., Bassi et al., 2012; Nissen-Lie et al., 2015), (k) extreme contexts such as rituals (Lee, 2013) and extreme weather during climbing (Bassi and Delle Fave, 2010) and even (l) first-aid activities, whereby professionals experienced more flow than volunteers (Sartori and Delle Fave, 2014). This large list shows that flow can occur in a large variety of activities and contexts (Diaz and Silveira, 2013).

Are there differences between activities in their likelihood to produce flow? In general, it was found that flow is higher during working-activities compared to (active and passive) leisure activities (Engeser and Baumann, 2016). For example, Bassi and Delle Fave (2012a) found that school teachers experienced more flow during work than during free-time (see: paradox of work; Csikszentmihalyi and LeFevre, 1989). The paradox of work states that although work is commonly associated as an unpleasant activity, individuals experience more flow—a pleasant state—during work than during free-time (Csikszentmihalyi and LeFevre, 1989). In contrast, MacNeill and Cavanagh (2013) found that school leaders experienced more flow in non-school contexts. Rodríguez-Sánchez et al. (2011b) found that the flow component enjoyment was higher during non-working activities whereas absorption was higher during working activities. Magyaródi and Oláh (2015) found that work, sports and creative activities were the most typical solitary activities and work and sports were the most typical social activities that foster flow. Of course, flow has also been investigated in social contexts (e.g., Ryu and Parsons, 2012). For a better overview, the authors of this scoping review decided to define “*interindividual factors*” as a separate category (see below). At work, planning, problem solving, and evaluative activities especially seem to foster flow (Nielsen and Cleal, 2010).

#### Contextual Factors and Their Relationships With Flow

The research explored in this scoping review shows that there are many contextual factors that are associated with flow at work. Maybe that is why Ceja and Navarro (2012) found in their study that there are many abrupt changes in experiencing flow at work; While flow is a self-reinforcing inner state of consciousness, contextual factors are external circumstances which cannot fully be controlled by an individual. A change of contextual factors can thus interrupt flow—and the more contextual factors exist that affect flow, the more likely are such sudden changes in flow. It was found that the motivating job characteristics of Hackman et al. (1975) are context factors that are positively associated with flow in the workplace (Demerouti, 2006; Maeran and Cangiano, 2013). In line with this, it was found that subjective relevance (Shernoff et al., 2003; Dawoud et al., 2015), importance (Rha et al., 2005; Engeser and Rheinberg, 2008), and meaningfulness (Banfield and Burgess, 2013; Hsu et al., 2013; Jonson et al., 2015; Bonaiuto et al., 2016) are positively associated with flow. All of these are concepts at the interface between person and context; if a context (e.g., a certain task or environment) aligns with the needs, values or motives of a person, it will become subjectively relevant, important and meaningful. Moreover, feedback and support are relevant precursors of flow (Bakker, 2005; Guo and Poole, 2009; Steele and Fullagar, 2009; Panadero et al., 2014; Swann et al., 2015a). Creative tasks (e.g., sketching: Cseh et al., 2016) or having the opportunity for creativity (Moneta, 2012) seems also to be positively associated with flow. Having a clear goal (Shin, 2006; Guo and Poole, 2009; van Schaik et al., 2012) and a clear role (Steele and Fullagar, 2009; Panadero et al., 2014) as well as having control (Shernoff et al., 2003) or autonomy (Bakker, 2005) are positively associated with flow. Furthermore, it was found that being prepared (Swann et al., 2012) and being recovered in the morning is positively associated with flow at work during the day (Debus et al., 2014). Smith et al. (2012) found that organizational safety climate is associated with flow. In general, having enough resources is positively associated with flow at work (Mäkikangas et al., 2010); a study by Emanuel et al. (2016) found that job resources (e.g., support from supervisor and autonomy) are positively associated with the flow experience of journalists. In addition, an internal locus of control was found to be positively associated with freelance journalists’ flow experience.

There are several factors of a game’s design that seem to facilitate flow. In general, interactivity and playfulness are positively associated with flow (Rha et al., 2005; Voiskounsky et al., 2005; Cheok et al., 2011; Hong et al., 2013; Khan and Pearce, 2015) in gaming and in the working or learning context (Dawoud et al., 2015; Meyer et al., 2016), while one study found that the content is more important for flow than the interaction (Marston, 2013). Sharitt (2010) additionally found that a balance of difficulty was an important criterion for flow-associated game design. Lastly, instruction type is also relevant for flow: Winberg and Hedman (2008) found in an experimental design that guided instructions foster the flow components of enjoyment and concentration whereas free guiding instructions facilitate the flow component of control.

#### Fit of Contextual Factors With Characteristics of the Individual

Besides general context factors, the fit of the context to the individual (see also *Personality and Flow*) seems to particularly matter: Moneta (2012) found evidence that a person-environment-fit fosters flow. In this respect, the best investigated flow condition is the fit between challenges of the activity and skills of the person, i.e., the challenge skill balance (Gnoth et al., 2000; Eisenberger et al., 2005; Engeser and Rheinberg, 2008; Freer, 2009; Bassi et al., 2012; Belchior et al., 2012; Hsu et al., 2013; Harris et al., 2017; ease of use; Voiskounsky and Smyslova, 2003; Keller and Bless, 2008; Katuk et al., 2013; Llorens et al., 2013; Wrigley and Emmerson, 2013; Koehn and Morris, 2014; Sartori and Delle Fave, 2014; Sartori et al., 2014; Wang and Hsu, 2014). In line with this, Schmierbach et al. (2012) found that the possibility to personalize a game facilitates flow. A study from Baumann et al. (2016) found that a dynamic (i.e., varying demands) and not a static challenge-skill balance is best for flow. Similar results were found by Ceja and Navarro (2009) who state that flow experiences follow a complex dynamic. In general, and in association with the challenge-skill balance, having enough resources (Delle Fave and Bassi, 2009; Bakker et al., 2011) and risk or uncertainty (Urmston and Hewison, 2014) are associated with flow. Another example for a flow-promoting fit between the context and the individual was shown by Vittersø et al. (2001), who found that a fit between individual’s preferred recreational mode and the recreational activity (e.g., being active or passive) was positively associated with flow.

### Interindividual Factors

The category *Interindividual Factors and Flow* included studies that dealt with flow in social contexts, measured at the individual or collective level and as a social phenomenon (e.g., team flow, group flow, social flow etc.). Studies which looked at the effects of flow on more than one individual (e.g., small groups, social settings, networks, and other collectives) were also included. Expert ratings revealed that twelve articles met these inclusion criteria. Another article was included by our experts, although they were not found in the literature search. The final list of articles that were integrated into this section is shown in Table 10.

**TABLE 10:** Interindividual factors.

Authors	
Bakker (2005) Bakker et al. (2011) Boyns and Appelrouth (2011) Gute et al. (2008) Keeler et al. (2015) MacDonald et al. (2006)	Magyaródi and Oláh (2015) Ryu and Parsons (2012) Salanova et al. (2014) Smith et al. (2012) van Schaik et al. (2012) Zumeta et al. (2016)
Added by the experts:	
Walker (2010)	

*Studies included dealt with flow in social contexts, measured at the individual or collective level and as a social phenomenon (e.g., team flow, group flow, social flow etc.). Also included were studies which looked at the effects of flow on more than one individual (e.g., small groups, social settings, networks and other collectives).*

Even though many human activities are done in social settings, the research on collective flow has not been vast, but the number of contributions is recently growing. As subtopics, we differentiate the experience of flow at the *individual level*, while being part of a social context (cf. Walker, 2010), from the experience of flow at the *collective level*, as if the collective has an experience of flow (cf. Sawyer, 2003).

#### Interpersonal Flow Studies at the Individual Level

Walker (2010) differentiates solitary flow experiences from social flow experiences, the latter varying on the degree of interdependence (ranging from co-active to highly interdependent). He found that participants in highly interdependent (sport) teams reported more joy than individuals performing less interdependently. Ryu and Parsons (2012) investigated social flow in the context of collaborative mobile learning and found that experiencing social flow is positively associated with the mobile learning experience. In addition, Bakker et al. (2011) studied team member flow experience among young soccer players. In short, the results indicate that social support and performance feedback from the coach are important facilitators of flow.

Magyaródi and Oláh (2015) found that for *interpersonal* flow experiences in social settings the level of perceived challenges should be high, as well as the level of cooperation, the immediateness/clarity of feedback, and the perceived level of skills. van Schaik et al. (2012) studied flow within an immersive virtual environment for collaborative learning. They found that the flow enablers challenge-skill match, goal clarity and feedback mediated the relationship between task constraints and learning experience. In the context of a group music composition task, MacDonald et al. (2006) found that the “no fear of failure” condition contributed to better flow. Moreover, they found that higher levels of flow related to a higher quality level of the output. In music teaching, Bakker (2005) found a crossover of the teacher’s experience of flow to students through contagion. In addition, Keeler et al. (2015) found that group singing reduces stress and fosters social flow at the individual level.

In the context of work, Smith et al. (2012) found that flow moderates the effect of leadership styles on job satisfaction and organizational commitment and partially mediates the effect on safety climate. Gute et al. (2008) found through the analysis of existing interview reports from highly creative persons that parents who foster *both* integration (e.g., providing emotional support) and its opposite, differentiation, (e.g., stimulation to work on personal goals) cultivate environments for creativity and flow. Using Csikszentmihalyi’s flow theory, Boyns and Appelrouth (2011) investigated the suspension of activity in public isolation and found that for most participants, “non-doing” leads to counterparts of the flow characteristics (e.g., boredom and anxiety).

#### Interpersonal Flow Studies at the Collective Level

Pioneering research in this perspective is the work of Keith Sawyer who defined group flow as *a collective state that occurs when a group is performing at the peak of its abilities* (Sawyer, 2003, p. 167). In this line, Salanova et al. (2014) found that collective efficacy beliefs predict collective flow over time, and that the two constructs are reciprocally related. Also, Zumeta et al. (2016) investigated *shared flow* during positive collective tambours/drummer (Tamborrada) gatherings. They found that positive collective gatherings stimulate shared flow experiences and in turn promote personal wellbeing and social cohesion.

### Cultural Factors

Culture can be seen both as an antecedent and as a consequence of flow experience. On the one hand, culture directs the individual toward actions, behaviors and activities that can more or less favor the experience of flow activities (Delle Fave et al., 2011); on the other hand, flow affects the actions of individuals, their decision-making processes, their focus of attention and their focus of behavior on certain purposes, which cause elements of culture (Inghilleri et al., 2014). Considering this premise in the category *Cultural Factors and Flow*, studies were included that did cross-cultural investigations or dealt with individualism or collectivism, culture and the construction of the self, social identity, or special artifacts (e.g., Manga). Additionally, studies that addressed specific countries were also included here. Expert ratings revealed that 13 articles met these inclusion criteria. Another three articles were included by our experts, although they were not found in the literature search. The final list of articles that were integrated into this section is depicted in Table 11.

**TABLE 11:** Cultural factors.

Authors	
Asakawa and Csikszentmihalyi (2010) Brown and Westman (2008) Busch et al. (2013) Delle Fave and Bassi (2009) Garces-Bacsal (2016) Guizzo and Cadinu (2016) Jonson et al. (2015)	Lee (2013) Liu et al. (2015) Mao et al. (2016) Mesurado et al. (2016) Niu and Chang (2014) Tanaka and Ishida (2015)
Added by the experts:	
Asakawa (2010) Coatsworth et al. (2005) Montijo and Mouton (2016)	

*Studies included did cross-cultural investigations on flow. Also included were studies that dealt with individualism or collectivism, culture and the construction of the self, social identity, or special artifacts (e.g., Manga). Additionally, studies that addressed specific countries were also included here.*

To understand the interaction between flow and culture, there are two main frameworks of research: the cross-cultural perspective, focusing on a comparison of flow experience between different cultures, and the cultural perspective, focusing on the role of flow in the diffusion or the maintenance of specific relevant cultural phenomena.

#### Cross-Cultural Perspective

Even if flow has been recognized as a universally valued subjective state (Asakawa, 2010; Delle Fave et al., 2011; Csikszentmihalyi and Wong, 2014), several studies collect data about cross-cultural differences in the flow experience (e.g., Garces-Bacsal, 2016). Results in this field seem not to be proposing a unique view about which kind of culture gives more opportunity to its members to experience flow. Despite studies finding higher frequency and intensity of flow in Western societies compared to non-Western ones (Asakawa, 2010; Liu et al., 2015; Mesurado et al., 2016), Western individuals seem to have a lower propensity to experience flow in meaningful social activities, related to future goals and linked to personal growth (Coatsworth et al., 2005; Asakawa and Csikszentmihalyi, 2010; Montijo and Mouton, 2016). Group activities involved with flow are associated with higher reports of social identification in collectivistic societies than in individualistic ones (Mao et al., 2016). Data shows that flow experience is more intense within the members of cultures characterized by a good balance between the values of both autonomy and relatedness (Busch et al., 2013).

#### Cultural Perspective

Flow seems to be involved in the spread and the maintenance over time of several specific cultural phenomena. Flow experience represents a useful concept to reach a deep knowledge of youth behavioral trends (Niu and Chang, 2014) and it seems to be involved in several leisure activities that are characteristic of different cultural environments (Jonson et al., 2015; Tanaka and Ishida, 2015). Furthermore, flow correlates with extrinsic and intrinsic religious orientations (Brown and Westman, 2008). An Italian study (Guizzo and Cadinu, 2016) demonstrated that flow disruption can depend on the degree to which people rely on society beauty ideals typically promoted by Western media. Further, flow can play a key role exerting influence on the quality of the migration experience (Delle Fave and Bassi, 2009; Lee, 2013). Despite the implication that flow can foster positive cultural and societal phenomena (Delle Fave and Bassi, 2009; Lee, 2013; Jonson et al., 2015), its amoral character can also lead to dysfunctional ones (i.e., Niu and Chang, 2014). Evidence in this area of interest are still scarce and further research is needed to clarify and validate results.

## General Discussion

With this Scoping Review, we aimed to (1) present a framework to structure flow research and (2) provide a systematic overview on empirical flow research of the years 2000–2016. In this general discussion, we summarize the results of this review, outline central points of discussion and describe strengths and weaknesses identified in the literature. Following this, we address our final aim: (3) to discuss the implications of our review for future research.

### Framework to Structure Flow Research

Firstly, we provided a framework to structure flow research. Secondly, this was then used to collate and summarize the existing literature in the field. Thirdly, based on the first and second, we are able to discuss implications for future research.

The framework distinguishes between individual, interindividual, contextual and cultural levels. Most research has been done on the individual level, with Personality (40 studies; Table 3), Motivation (54 studies; Table 4), Emotion (49 studies, Table 6), Cognition (26 studies; Table 7), and Behavior (53 studies; Table 8) being the largest categories. On the individual level, the Physiology of flow (21 studies; Table 5) is the least studied category; however, in recent years there is a growing trend of research being conducted in this area. There are 94 context level studies (Table 9). In comparison, research on flow at the interindividual (13 studies; Table 10) and cultural level is underrepresented (16 studies; Table 11).

### In a Nutshell: Discussion of Findings Within the Categories

#### Personality

The personality studies on flow were divided into four categories: autotelic personality, dispositional proneness to experience flow, flow and motive-fitting situations and other motives and personality traits. Several dimensions seem to characterize the concept of autotelic personality which are related to flow. However, there is still no widely agreed upon definition of the autotelic personality. Studies on individual differences in flow experiences depend on both situational variables, e.g., the environmental opportunities to engage in flow promoting activities, and personality traits (i.e., openness to experience, extraversion, and conscientiousness). Situational factors seem to have a stronger influence on flow experiences (Fullagar and Kelloway, 2009; Ullén et al., 2016). However, more research is needed to specify the relationship of dispositional and situational factors to predict flow experiences.

Achievement motives and other motives and personality traits, (i.e., optimism, autonomy, self-handicapping, self-control) also seem to be associated with flow experience. However, the variety and even inconsistency (e.g., shyness and mental toughness) of personality traits and motives associated with flow, make it difficult to draw overall conclusions. Relating personality traits and motives to fitting situations seems to be a more promising way to investigate the effects of personality traits and motives on flow in the future.

#### Motivation

Flow experience is historically linked to motivation (see e.g., Heutte et al., 2021). In line with this, results of this category showed that many motivational indicators, such as volition, engagement, goal orientation, achievement motive, interest, and intrinsic motivation are positively related to flow. Flow was also investigated in the context of self-determination, with results showing associations of flow with autonomous and controlled motivation. Results thus indicate that flow can be considered one of the major volitional theories. This is also in line with results of a meta-analysis by Fong et al. (2015), that highlights the links between flow antecedents (e.g., concentration, merging of action and awareness, and feedback) and sense of autonomy, one of the central components of self-determination. Finally, self-efficacy was an often investigated motivational concept, with results confirming a relationship between self-efficacy and flow. While first studies in this category were largely correlational, more recent studies have started to investigate models that integrate various motivational concepts (often from Bandura or Deci and Ryan’s theories) as predictors of flow using structural equation models.

A new and promising challenge in the category *Motivation* concerns modeling research studies that investigate both collective motivational conditions and social dimensions of flow (see Salanova et al., 2014; Heutte et al., 2016). However, in order to fulfill this aim, this work requires construction and validation of multidimensional and short, specific measurement instruments for flow, which also include collective motivational dimensions of flow.

#### Physiology

Studies on the physiology of flow are yet in their infancy and results are scarce and inconsistent. While the first studies in this category were mostly correlational, more recent studies have started to investigate flow using experimental designs. Some studies regard flow as a predictor of certain physiological states. Others regard physiological states as predictors of flow. A clear physiological pattern of flow has not yet been identified, but this seems to be the next major task for research on the physiology of flow. Presumably, the physiological pattern during flow will not be represented by a single physiological indicator, but rather by a combination of several different physiological indicators. Current developments of machine learning may help to identify such a pattern. Once a physiological pattern of flow is identified, this will help flow research to find a deeper understanding of the flow concept. Flow can then be measured continuously during an activity, without the need to interrupt people. Accordingly, the dynamics of flow over time can be assessed, as well as the variations of flow intensity. Still, it is unlikely that there will be just the one flow-characteristic pattern; rather the physiology of flow depends on the particular activity that one is doing, with people in flow showing the optimal physiological activation to meet task demands (see Peifer, 2012). Building upon this, the second future research question is how context conditions, such as characteristics of the task (e.g., difficulty) or conditions at the interface between context and person, (e.g., task relevance) moderate the typical physiology of flow.

#### Emotion

Studies under the topic of emotion and flow cover a wide range of concepts and variables related to affect, wellbeing, or specific feelings like enjoyment. In general, results show a clear association between flow and positive emotional states. There is clearly a predominant focus on the study of *positive* affect, with only few studies analyzing the relationship between negative affect and flow, so more research is needed here. The majority of the studies investigated the role of flow as a predictor of different emotional aspects, showing that the reversed relationship is less studied. Flow and related emotional aspects have been studied mainly from an individual or subjective perspective, with social components of flow and emotion becoming an emergent research issue. Studies under this topic shed light on the importance of understanding the emotional functioning of flow experience to improve its positive outcomes in individuals’ lives. Results of the various studies show a large spectrum of practical implications in different areas, such as sports, educational contexts, the video game industry, organizational areas, general health, or quality of life.

#### Cognition

Cognition studies on flow are extremely broad and touch on very different topics. Most of these look at flow in specific fields and include some cognitive variables but without a main focus on them and also without deeper discussion of the cognitive aspect of flow. “Attention” appears in several “cognition and flow” studies, but how flow and attention exactly are linked is not sufficiently explained. For example, some studies point to attention skills as a necessary precondition for obtaining flow, whilst other studies find that sometimes, people with poor attention skills can still find flow in, for example, activities where they have high levels of interest and engagement. More research is needed to understand the relation of flow with cognitive processes. This research could also help both deepening and widening some of the research questions that have emerged from the reviewed studies, relating, for example, to the disassociation between sense of control and sense of agency in flow experiences, or the understanding of the exact role of awareness in optimal experience.

#### Behavior

Overall, many effects of flow on behavioral outcomes were shown. Most studies in this category dealt with performance-related outcomes and found positive association between the two. However, one has to be careful when interpreting direction of the effects: Most studies in this category are correlational only and therefore it is not possible to deduce the direction of effects. Landhäußer and Keller (2012) argue that flow, on the one hand, has a direct positive effect on performance, because individuals in flow are highly concentrated. On the other hand, individuals have a higher motivation to re-engage in a task when flow was experienced resulting in higher performance through practice (Landhäußer and Keller, 2012). Accordingly, there is a clear need for longitudinal studies and for identifying moderators and mediators in the relationship between flow and performance in order to specify the direction of effects. Other studies looking at behavioral outcomes such as customer-oriented behavior and (online) consumption-behavior hold interesting implications for organizations, advertisement and therapy, though again more longitudinal and experimental research should be conducted to reach more solid conclusions and to start designing useful interventions to increase performance and wellbeing.

#### Contextual Factors

In summary, flow occurs in many different contexts and activities, and there are many contextual factors that promote flow. A fit between contextual factors (e.g., demands) and individual characteristics (e.g., skills; see also section on personality and flow) seems to play a particularly important role in the emergence of flow. However, this category contains many articles, as it includes all environmental factors which may affect flow experience. It is presumably the broadest category within this review. While we have at least distinguished the social environment as a sub-category within the contextual level, future frameworks could further distinguish different environmental factors, such as factors on the task level, the social/organizational level (for work settings) and factors at the interface of the individual with the task and the organization. A framework which has recently tried to implement such a structure is the three spheres framework of flow antecedents (Peifer and Wolters, 2021). In addition, it could be useful to differentiate direct interaction from more indirect social influence such as organizational climate.

#### Interindividual Factors

Overall, studies in this category were yet quite scarce, but we could see a growing tendency to measure, conceptualize and investigate interindividual factors of flow. This is evidenced by a growing number of studies published in more recent years within the timeframe of our review. Furthermore, within the EFRN, we see a growing number of conference contributions and EFRN members starting to investigate flow in social contexts. We conclude that there is increasing awareness of interaction effects among people in relation to flow experiences. When reviewing the existing literature, we found that the research on interpersonal flow lacks a broad conceptualization and is instead limited to individual flow experiences while being part of a collective (e.g., dyad, group). A clear challenge of future flow research is to differentiate individual flow in social contexts from social flow as a social phenomenon with potentially different qualities than individual flow. A recent suggestion to differentiate flow and team flow was made by Peifer et al. (2021), suggesting that flow and team flow share the central components of individual flow, while team flow-specific components are added. In their studies, van den Hout et al. (2018) bridge individual experiences of flow with collective experiences of flow. In their conceptualization of team flow, they differentiate individual experiences of flow while being part of a team dynamic, with experiences of flow at the team level, where the team dynamic (or team process) itself, as a coherent unit, is *flowing*. When all members that are part of the team dynamic are experiencing flow while executing their personal tasks/roles for the team, and the collective itself is flowing a unique experience emerges, which they refer to as *full team flow*, that is originated by seven prerequisites and four experiential characteristics (van den Hout et al., 2019; van den Hout and Davis, 2021).

Future directions include studying interindividual flow through the grounded theory approach (see Csikszentmihalyi, 1975), conceptual cross-fertilization with social and organizational psychology, and developing reliable self-reported and behavioral measures of the phenomenon, experimentation and longitudinal studies. Social flow and its emotional features appear as an emergent issue in flow studies. However, finding a measure for assessing interindividual flow as a group phenomenon without passing through aggregation of self-reported individual data is a major methodological challenge for future research of this topic.

#### Cultural Factors

*Culture and Flow* represents an important theoretical perspective and several theoretical and empirical contributions in this field have been collected recently in specific scientific books (i.e., Delle Fave et al., 2011; Csikszentmihalyi and Wong, 2014; Inghilleri et al., 2014). Despite this, we notice a general lack of published empirical articles dealing with flow in the cultural context, even if existing research shows its underlying relevance for investigating flow-fostering activities. Furthermore, flow has the potential to interact significantly with cultural phenomena of different nature, both positive and negative for human beings. Thus, we suggest that future research should put additional emphasis on the effects of culture on flow and vice versa.

### Overarching Aspects for Future Research and Limitations of This Review

After having discussed the specific open research questions for each category, we would now like to outline general aspects for future research, which we could identify as overarching topics and as limitations of this review. In particular, these concern (1) definitional and operational issues, (2) methodological issues and the resulting problems of causal conclusions regarding antecedents and consequences of flow, as well as (3) the time frame of this scoping review.

#### Definitional and Operational Issues

Many studies worked with different definitions and operationalization of flow experience so one must be careful when comparing results. For example, some studies (e.g., Baumann and Scheffer, 2011; Oertig et al., 2014) used the Flow Short Scale (Rheinberg et al., 2003). Others used the Practice Flow Inventory (Heller et al., 2015), Jacksons’ and Eklunds’ (2002) Dispositional Flow Scale-2 (Sinnamon et al., 2012) the Flow State Scale-2 (Wrigley and Emmerson, 2013) or the EduFlow model (Heutte et al., 2016). While beyond the scope of this review, for future research, there is a need to find a common definition and operationalization of the flow concept, including a common measure of flow which is used in future research to enhance the comparability of results. The EFRN has started to fulfill this aim by agreeing on a definition of flow (see section “Introduction”), and members of the EFRN have suggested models to aggregate components of flow and team flow (e.g., van den Hout et al., 2018; Heutte et al., 2021; Peifer and Engeser, 2021; Peifer et al., 2021). The next steps will be to discuss and agree on models and respective measurements.

#### Methodological Issues

In general, while conducting the review, the authors found many correlative studies, and causal interpretation of such data is not possible. Many of the reported studies suggest a causal interpretation of their results based on theoretical assumptions. However, this is problematic, as different theoretical assumptions also seem possible. In conclusion, antecedents and consequences of flow are not yet as clear as they should be, considering the immense amount of studies which have been conducted. While this is beyond the scope of this review, future reviews should focus on a systematic look at the methods behind the studies. Here, we want to emphasize that what is needed in the future is mainly longitudinal and experimental studies.

Another methodological aspect which we found as an overall topic is that most of the research was conducted with (young) adults; there is a lack of flow research on children as well as adolescent and elderly populations. In general, there is a need for studies testing more complex models to understand multiple relations between variables.

#### Time Frame and Inclusion Criteria of This Scoping Review

Our Scoping Review provides a systematic overview on flow research between the years 2000 and 2016. A task force of flow research from the EFRN united their expertise in order to provide a sound scientific summary and discussion of flow research in these years and implications for future research. The work on this scoping research started in November 2015, during the EFRN meeting in Braga, Portugal. The literature search was conducted in 2016 and updated in 2017 in order to cover all articles until the end of the year 2016. The process of writing and revising the article took a long time and another update of the literature search would have exceeded the word limit of a journal article, particularly as flow research has been further increasing in more recent years.

Furthermore, we set strong exclusion criteria by only allowing studies that mentioned “*Csikszentmihalyi”* and that were listed in specific search platforms. We selected the most relevant platforms for our literature search, thereby excluding other platforms (e.g., CINAHL, ProQuest, SocIndex, and SocAbs). Therefore, it is entirely possible that not all relevant flow studies are included in our review. As experts were allowed to add additional studies they considered relevant, we hope that in the final analysis we have identified the majority of relevant studies. Furthermore, we only included studies that were published in the English language, and there are certainly interesting results published in other languages that are not covered here.

While the time frame as well as the strong exclusion criteria are clear limitations of this review, we still believe that the provided overview will help to stimulate and direct future flow research.

## Conclusion

Flow research between 2000 and 2016 has made huge progress in understanding flow. Our review provides a framework to cluster flow research and gives a systematic overview about existing studies and their findings in the field. While much research has been done in the past, our review derives future lines of research to foster scientific progress in flow research.

## Author Contributions

CP: project coordinator, introduction and discussion. GW: project coordinator, literature research, discussion, behavior, and context factors. ST: professional advice during the process. GW and LH: personality. JHe and GW: motivation. CP and JT: physiology. TF, DT, and CF: emotion. ST and FA: cognition. JHo and MŠ: interindividual factors. LP: cultural factors. LC: review of parts of the manuscript. All authors: categorization and selection of abstracts.

## Conflict of Interest

The authors declare that the research was conducted in the absence of any commercial or financial relationships that could be construed as a potential conflict of interest.

## Publisher’s Note

All claims expressed in this article are solely those of the authors and do not necessarily represent those of their affiliated organizations, or those of the publisher, the editors and the reviewers. Any product that may be evaluated in this article, or claim that may be made by its manufacturer, is not guaranteed or endorsed by the publisher.
